# Aviation safety training methodology

**DOI:** 10.1016/j.heliyon.2021.e08511

**Published:** 2021-11-29

**Authors:** Krasin Georgiev

**Affiliations:** Department of Aeronautics, Technical University of Sofia, 8 Kl. Ohridski Blvd, Sofia, Bulgaria

**Keywords:** Aviation safety, SMS, Safety training, Risk models

## Abstract

Safety management systems (SMS) are widely accepted as a way of improving safety and are currently required by the aviation regulations. Safety training and education of all professionals in the aviation safety system is vital for the success of any safety management strategy. At the same time some of the terms in the field are not uniquely and consistently defined, e.g. hazard, threat, safety issue, risk, risk area to name a few. This could be confusing for the users of the SMS who are not required to be safety experts. In addition, the abstract nature of the safety concepts and system thinking combined with the formal language of the regulations makes catching and keeping the attention of the trainees a challenge.

A methodology for aviation safety training was developed and studied. The first objective of the elaboration was to introduce the students gradually by building on tangible and actionable items as performance indicators and accident scenarios. The second objective was to connect the above material with the standard safety management framework as defined by the International Civil Aviation Organization (ICAO). The main emphasis was on understanding the fundamental concepts, notations and relationships through practice. The presented approach has demonstrated higher engagement and improved material mastery of the students.

## 1 Introduction

Safety management systems (SMS) are widely accepted as a way of improving safety and are currently required by the aviation regulations [1]. At the same time the corresponding guidelines are quite general [2], [3]. Proper implementation of safety management depends heavily on the expert knowledge of the safety officers and frontline staff at each organisation and state administration.

It could be argued that some of the notions in the field are not uniquely and consistently defined. Terms like hazard, threat and safety issue are used as initiating events, latent factors or specific operations [2], [4], [5]. The safety risks are expressed as outcomes, risk areas, threat categories, accident groupings, etc. [3], [5], [6], [7]. This is not surprising as more then eight hundred methods, techniques, models and databases used for safety assessment are available and their number is increasing [8].

Safety is hard to measure, hard to quantify, hard to verify and to validate in the field. Some deficiencies in current risk modelling of air traffic are discussed in detail in [9], [10]. Maintenance and operational issues are not scientifically attractive (author's view). If experts struggle to set the scene with common understanding of the basic concepts and terminology, what could be expected from the users from unrelated disciplines?

Another issue specific to training is that it is hard to catch and keep the attention of the trainees with too general and too abstract ideas as the ones in system safety. The formal and verbose language of the regulatory documents is also a barrier to a detailed study of the material. This issue can be handled by applying a modern teaching principle of “playing the whole game” [11]. This means to provide practice with a simple but still working version of the system.

The success of safety management depends on the active involvement of all participants in the system. To quote Donald Pitts's thoughts on the Heinrich's accident pyramid “The largest body of information lies in the unreported occurrences which, for the most part, go unnoticed except by those who personally experience the event.” [12]. Failure to identify a hazard by the frontline staff means that safety professionals are limited in what they can model and assess.

The issues highlighted above combined with the limited literature and guidance on the specifics of the aviation safety training open an area appropriate for scientific research. Safety training and education of all professionals in the aviation safety system is vital for the success of any safety management strategy. It is actually a required element of the safety promotion part of the SMS.

A methodology for aviation safety training was developed following the view of learning by wholes [11]. The first objective of the methodology design was to introduce the students gradually by building on tangible and actionable items as performance indicators and accident scenarios. The second objective was to connect the techniques learned with the standard safety management framework as defined by the International Civil Aviation Organization (ICAO) thus providing a framework capable to accumulate empirical knowledge from the edge (the practitioners on the front line). Finally the methodology was proved by measuring its effectiveness in teaching university students.

## 2 Methodology for aviation safety training

Three perspectives of aviation safety were included in the training syllabus following the criteria for tangible and actionable items that represent the whole system. Aviation domain knowledge combined with data analytics provide the first “exploratory” level of safety understanding – the performance indicators. Modelling chains of events is the second “causal” level of safety understanding. Cause effect relationships are explicitly modelled by following the rules of logic and probabilities. Mastering the hazard and risk concepts is completed in the third “system” level of the methodology. It provides the framework for managing the safety in a real world where data are often not enough and casual relationships are too complex to be explicitly modelled.

### 2.1 Event types and safety performance indicators

Safety performance indicators are measures of the level of safety achieved. The following tasks help to develop the corresponding knowledge and skills:

– building and clarifying of notations;
– practising definition and calculation of indicators with simple dataset;
– practising calculation of indicators using real data;
– reviewing examples from safety reports from industry, governments and international organisations.

The terminology for different event types is introduced following the international standards [13]. Accidents, incidents and other occurrences are defined based on event attributes as aircraft damage and highest injury level. The severity of the actual and potential consequences is discussed. Practising defining, symbolic representation and calculation of performance indicators allows the student to understand and self-discover:

– What are “Safety Performance Indicators”?
– What are “Safety data” and where to find them?
– Taxonomies – classification of events, causes and factors
– How to define the indicators – counts or ratios; serious or minor events; leading or lagging; generic or specific
– How to present the indicators (visualizations).

An example safety case starts with the list of reported events in Bulgaria for the year 2006 (Table 1, columns 1 to 5) and the corresponding volume of flight activity [14]. For the period from 01.01.2006 to 31.12.2006 there were 98465 flight hours and 57873 departures in commercial aviation; 2497 flight hours and 6697 departures in the general aviation; 3324 flight hours and 14498 departures for aerial works. These data are sufficient for a range of exercises.

**Table 1:** List of accidents (columns 1 to 5) [14]. Event classification (columns 6 to 8) is the first step in the analysis.

Date	Aircraft	Operation	Fatalities	Cause	OP^a^	E^b^	CF^c^
1	2	3	4	5	6	7	8

04.03.2006	An-12	Regular cargo	0	Landing gear collapse	**C**	**ACC**	**AT**
11.04.2006	An-2	Agricultural	2 of 3	Flight crew error	**A**	**F**	**HF**
11.04.2006	Aviatika 890CX	Agricultural	1	Flight crew error	**A**	**F**	**HF**
05.08.2006	P2002JF	Training	0	Flight crew error	**G**	**ACC**	**HF**
28.08.2006	Ka-32	Fire fighting	0	Engine failure	**A**	**ACC**	**AT**

a OP (type of operation) – C (commercial aviation), A (aerial work), G (general aviation);

b E (type of event) – ACC (accident), F (fatal accident);

c CF (cause factor) – AT (technical), HF (human), ENV (environmental).

*Event classification.*  Each event is classified according to preselected criteria as shown in the last three columns of Table 1. The notations for the criteria and the possible categories used are

– by event type (*E*) – accident (ACC), fatal accident (F);
– by operation type (*OP*) – commercial aviation (C), aerial work (A), and general aviation (G);
– by cause factor (*CF*) – technical (AT), human (HF), and environmental (ENV).

*Indicators definition and calculation.*  After the initial event classification the indicators are defined as either a number of events of interest or as ratios. Introducing systematic notations is important for proper record keeping. The indicators included in Table 2 are defined using the following convention:

• Counts, general – $n_{E}$, e.g. $n_{ACC}$, $n_{F}$;
• Counts, per factor – $n_{E}^{\mathrm{CF}}$, e.g. $n_{ACC}^{\mathrm{AT}}$, $n_{F}^{\mathrm{HF}}$;
• Ratios, events per *M* units of work *W* – $K_{W,E}=M\cdot n_{E}/W$, where $M=10^{5}\ldots 10^{8}$, W∈{T,N,L,Z,LZ}, *T* – flight hours, *N* – departures, *L* – distance flown, *Z* - passengers. E.g., the number of fatal accidents per 10^5^ flights is $K_{N,F}=10^{5}n_{F}/N$;
• Ratios, average work *W* per event – $W_{avg,E}=W/n_{E}$, e.g. $T_{avg,ACC}$ denotes the average flight hours per accident.
• Ratios, per factor

**Table 2:** Safety performance indicators defined and calculated based on Table 1.

Operation	T	N	*n*_*ACC*_	*n*_*F*_	$n_{F}^{\mathrm{AT}}$	*K*_*T*,*ACC*_	*K*_*N*,*F*_	*T*_*avg*,*F*_	$N_{avg,F}^{\mathrm{HF}}$
Commercial	98465	57873	**1**	**0**	**0**	**1.0**	**0**	–	–
General	2497	6697	**1**	**0**	**0**	**40.0**	**0**	–	–
Aerial works	3324	14498	**3**	**2**	**0**	**90.3**	**13.8**	**1662**	**7249**
Total	104286	79068	**5**	**2**	**0**	**4.8**	**2.5**	**52134**	**39534**

Table 2 shows just a few of the many possible indicators that can be calculated with the data in Table 1.

*Practice with industry data.*  A dataset can be prepared by downloading publicly available data spreadsheets from the National Transportation Safety Board (NTSB) [15]. Considering a five year period from 2013 to 2017 there are 6590 safety records over more than 200 million flight hours. Each record is characterized by 21 attributes. A small sample from the dataset with a limited number of attributes is shown in Table 3.

**Table 3:** Sample from NTSB accident and severe incident dataset [15]. Some of the abbreviations are explained under the table.

ntsb no	ev date	highest injury	damage	FAR Part^a^	oper sched	acft categ	type fly	CICTT Event^b^	CICTT Phase^c^
CEN14FA141	2014-02-16	FATL	SUBS	091		AIR	INST	CTOL	TOF
WPR15CA073	2015-01-01	MINR	SUBS	091		AIR	PERS	LOC-I	TOF
WPR15CA074	2015-01-03	NONE	SUBS	091		AIR	PERS	ARC	LDG
ERA15FA088	2015-01-02	FATL	SUBS	091		AIR	PERS	FUEL	ENR
ERA15CA089	2015-01-02	MINR	SUBS	091		HELI	PERS	ARC	LDG
ERA15LA090	2015-01-03	MINR	SUBS	135	NSCH	AIR		SCF-PP	APR
CEN15CA097	2015-01-05	NONE	SUBS	091		AIR	PERS	LOC-G	TOF
CEN15LA098	2015-01-04	SERS	SUBS	091		AIR	PERS	LOC-I	MNV
ERA15FA096	2015-01-10	FATL	DEST	091		HELI	INST	LOC-I	APR
CEN15LA140	2015-02-09	MINR	SUBS	121	SCHD	AIR		ARC	LDG
SUR15CA002	2015-02-11	SERS	NONE	121	SCHD	AIR		CABIN	ENR
DCA15FA073	2015-02-24	SERS	NONE	121	NSCH	AIR		SCF-NP	STD
ERA15LA174	2015-03-31	SERS	SUBS	091		AIR	BUS	CFIT	APR

a The code for the Federal Aviation Regulation under which the aircraft flight was conducted – 091 (General Aviation), 121 (Air Carriers), 135 (Commuter and On-Demand Carriers);

b Event type code following CICTT occurrence categories taxonomy [16], e.g. CTOL (Collision with obstacle during Take-off and Landing), LOC-I (Loss of Control – In-flight), FUEL (Fuel related), SCF-PP (System/Component Failure or Malfunction (Powerplant)), CFIT (Controlled Flight Into or Toward Terrain);

c Flight Phase [16], e.g. STD (Standing) TOF (Takeoff), ENR (En route), MNV (Maneuvering), APR (Approach), LDG (Landing).

Understanding the information in Table 3 require some degree of aviation domain knowledge. Aviation operations are governed by State regulations, e.g. Federal Aviation Regulations (FARs) in the United States. The applicable regulations are given in column “FAR Part” and provide information about the type of operation. Some NTSB data are encoded using the CAST/ICAO Common Taxonomy Team (CICTT) taxonomy [16]. This includes occurrence categories (column “CICTT Event”) and phase of flight (column “CICTT Phase”). Other attributes shown in the table are the category of the involved aircraft (column “acft categ”), the primary purpose of the flight for general aviation aircraft (column “type fly”), scheduled or non-scheduled operations for air carriers (column “oper sched”).

**Figure 1:**
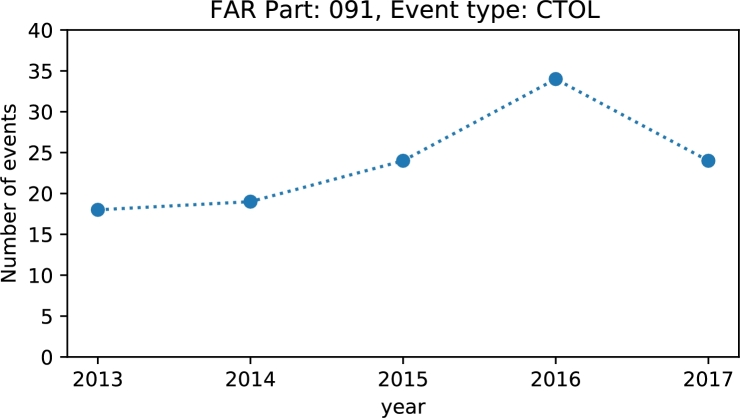
Line plot of the number of collisions with obstacle(s) during take-off and landing (CTOL) for general aviation operations (FAR Part 91).

**Figure 2:**
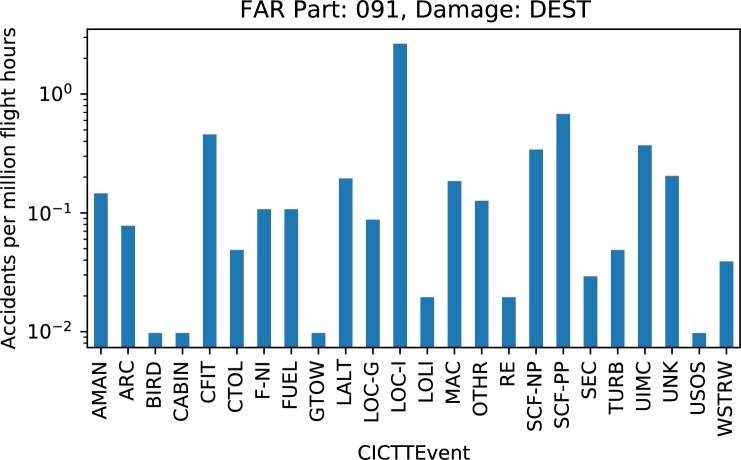
Distribution of accidents by CICTT occurrence categories [16] for FAR Part 91 operations and destroyed (DEST) aircraft.

Exploring public datasets provides context and motivation to self-discover and practice:

• decode main abbreviations by checking industry accepted taxonomies for occurrence categories, flight stages, consequences, etc. The classification schemes of keywords and definitions provide a “safety language” that is essential in safety information systems [17];
• calculate indicators assigned by the instructor or self-defined. A large variety of indicators can be defined and calculated as the number of categories and their combinations is significant;
• plot results by year, occurrence type, flight phase, etc. and make conclusions about trends, distributions and relationships (Figs. 1 and 2).

*Review industry reports.*  Many industry and government reports by ICAO, EASA, Boeing, Airbus, etc. present analysis and visualisations of safety statistics and indicators [6], [18]. Studying such materials can help the student to get confidence and ideas for future work.

### 2.2 Sequence of events, probabilities and outcomes

**Figure 3:**
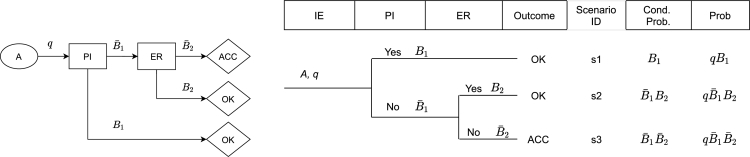
Simple Event Sequence Diagram, the equivalent Event Tree and scenario quantification.

The concept of accident scenarios and accident quantification is introduced through Event Tree (ET) and Event Sequence Diagram (ESD) models. An event tree is shown in the central part of Fig. 3 and the equivalent ESD is shown in the left part of the same figure. The initiating event (IE) is denoted by *A*, its probability by *q*, the pivotal events are successful “pilot intervention” (PI) and successful “emergency recovery” (ER). The conditional probabilities for the success and failure branches of the tree are denoted as $B_{i}$ and $\overset{¯}{B_{i}}$ respectively.

The following notations are explained in details for the simplest case of two events, e.g. event *X* is “wind shear”, and event *Y* is “loss of control”:

• Unconditional probabilities P(X) and P(Y) to have event *X* or event *Y* on is own;
• Conditional probability P(Y|X) for event *Y* in case of event *X*;
• Joint probability P(X,Y) of having both events *X* and *Y*.

An example practical exercise is to define the symbols $q,B_{1},{\overset{¯}{B}}_{1},B_{2}$, ${\overset{¯}{B}}_{2}$ used in Fig. 3 in terms of the notations in the list above. Experience shows that this task is not trivial even for students with formal education in probabilities and statistics mathematics. The expected answer for the ET on Fig. 3 is:

• q=P(A) – unconditional probability of the initiating event *A*;
• $B_{1}=P(PI|A)$ – conditional probability of successful PI if condition *A* exists;
• ${\overset{¯}{B}}_{1}=P(\bar{PI}|A)$ – conditional probability of $\bar{PI}$ (unsuccessful PI) if condition A exists;
• $B_{2}=P(ER|\bar{PI})$ – conditional probability of ER if pilot intervention was not successful;
• ${\overset{¯}{B}}_{2}=P(\bar{ER}|\bar{PI})$ – conditional probability of $\bar{ER}$ (unsuccessful ER) if $\bar{PI}$;

The right-hand side on the same figure is used for scenario and outcome quantification. This involves application of the product rule and the sum rule [19]:

(1) P(X,Y)=P(X)⋅P(Y|X)

(2) $$P(s_{1}\;\mathrm{or}\;s_{2})=P(s_{1})+P(s_{2})$$

where *X* and *Y* are pairs of events; $s_{1}$ and $s_{2}$ are mutually exclusive events or scenarios.

Considering the simple example on Fig. 3, practice is assured as:

• the product rule (1) is applied for each scenario and presented in columns titled “Cond.Prob.” and “Prob”. It is used to calculate the probability of an accident
  (3) $$P_{ACC}=P(s_{3})=q{\overset{¯}{B}}_{1}{\overset{¯}{B}}_{2}$$
• the sum rule (2) is used to calculate the probability of an outcome involving several scenarios, e.g. the conditional probability for favourable outcome “OK” is the sum of the conditional probabilities of both scenario 1 and 2
  (4) $$B_{OK}=B(s_{1})+B(s_{2})=B_{1}+{\overset{¯}{B}}_{1}B_{2}$$

**Figure 4:**
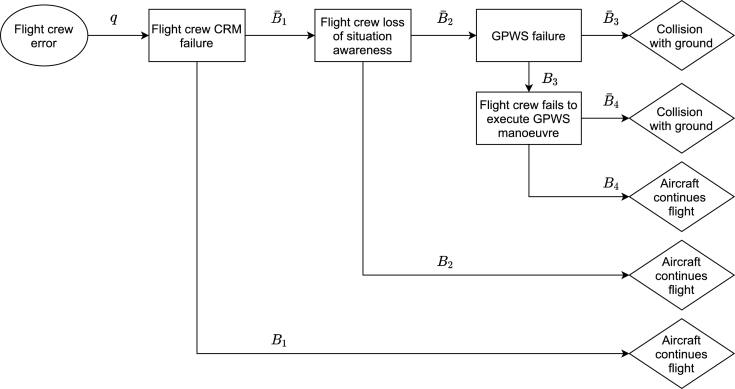
Accident scenarios for controlled flight into terrain (source: [20]). CRM – Crew Resource Management, GPWS - Ground Proximity Warning System.

Both quantitative and qualitative aspects of modelling can be further developed by studying existing models prepared by industry and research organisations. Generic accident scenarios for an integrated aviation safety model are available as part of a study prepared for FAA/IVW [20]. Such scenarios illustrate and help to differentiate the initiating events/threats, the pivotal events/controls, and the end states (outcomes) for specific accident types or risks. As an example the risk of controlled flight into terrain (CFIT) is presented in Fig. 4 after [20]. The emphasis is on understanding the casual nature of the accident and the role of preventive controls. Scenario and outcome quantification completes the ET and ESD practice but more advanced courses can include base event quantification also. A sense of the difference between initiating event frequency (hazard exposure) and potential accident probability should be developed.

### 2.3 Safety concepts and risk assessment

The abstract concepts of safety, hazard, and risk are reintroduced based on the ICAO definitions [1], [2]. Hazard is “A condition or an object with the potential to cause or contribute to an aircraft incident or accident.” Risk is “The predicted probability and severity of the consequences or outcomes of a hazard.” Safety is “The state in which risks associated with aviation activities, related to, or in direct support of the operation of aircraft, are reduced and controlled to an acceptable level.” These definitions should be recapped after some practice with safety metrics and event sequences is accumulated.

Studying alternative definitions, representations and examples is of key importance. The hazard definition can include event or circumstance [21] and activity [4], [5]. It is closely related to the corresponding cause and contributing factors for accidents and incidents. A local implication of one hazard or a combination of hazards in one part of operation become a “safety issue” [4]. Hazard can be part of normal operations or environment. “A possible direct cause that will potentially release a hazard by producing a top event” is called a threat [5]. The term “safety issue” is used in EASA Reports without definition and could include all of the above considerations [6]. Hazard should not be confused with hazardous outcome (consequences) or hazardous failure condition.

The classic view of the risk is that of a set of triplets <scenario, likelihood, consequences> [22]. Some publications treat the risk more like an event, a potential outcome that is assigned likelihood and severity ([3], [21]). Others treat the risk as a probability of an accident. The risk can be qualitatively represented with the following expressions (barrier model, risk profile, structural view, risk event):

• RISK = HAZARD / BARRIERS
• RISK = LIKELIHOOD x CONSEQUENCES
• RISK = HAZARD x VULNERABILITY
• RISK = HAZARD → EVENT → OUTCOME

The safety concepts practical exercise includes application of the terminology for a specific event. Let's take a lightning strike as an example. What is the hazard? – Thunderstorm, cumulonimbus clouds, other clouds? Forecast or proximity? What is the risk event? – Lightning strike, or damages from lightning (structural damages, instruments failure, electrical power failure, fuel fire, etc.). What is the severity of the consequences? The choice will depend on the objective of the analysis and the modelling approach to be used.

The safety modelling approach varies for tightly and loosely coupled systems [9], [10]. Description of all possible scenarios is feasible only for tightly coupled systems with a limited number of key failure modes and “programmable” operations. For loosely coupled systems the safety is provided through a “structure of defensive layers” that “filter out potentially hazardous situations” [10]. The potential failure modes are not explicitly enumerated and the risk can be determined based on the barrier performance.

The risk assessment practical exercises include studying representative risk assessment techniques, discovering the main components of the risk and clarifying the questions in Table 4. Three models were selected based on their wide adoption in aviation. These are outlined below together with their most attractive features.

**Table 4:** Risk related questions for reflection.

Consequences of what?
– The worst case scenario
– The most credible outcome
– The worst credible scenario
(the worst possible realistic scenario,
the worst foreseeable situation)

Probability of what?

– The safety event
– The potential outcome
– The remaining barriers failure

**Figure 5:**
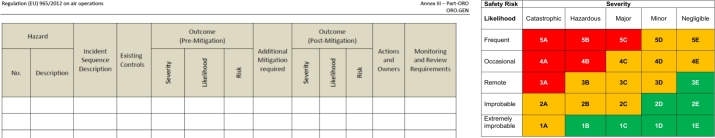
Hazard log and risk matrix.

*Hazard log and safety risk matrix.*  The operational experience related to existing and emerging hazards, threats or issues is systematically collected and analysed in terms of potential outcomes severity and likelihood, implicitly accounting for existing and proposed preventive and mitigating barriers. The hazard log documents the risk analysis in a tabular form (see Fig. 5, left). The criteria for consequence, severity and risk categories are predefined. Typical risk matrix is shown in Fig. 5, right)

• Recommended in ICAO Manual [2]
• Simple qualitative assessment ideal for small organizations

**Figure 6:**
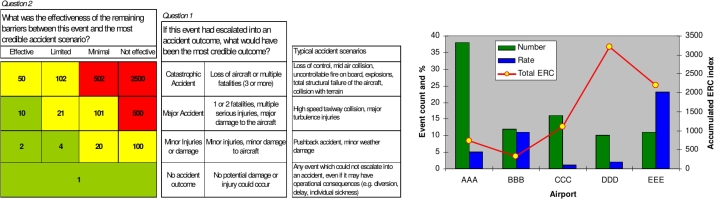
ERC matrix and ERC risk index [4].

*ARMS methodology.*  Safety occurrences and safety issues are analysed separately using the so called Event Risk Classification (ERC) (Fig. 6) and Safety Issue Risk Assessment (SIRA) methodologies (5) [4]. Barriers are modelled by assigning probabilities $B_{\mathrm{Avoid}}$ for avoiding the undesirable operational state and $B_{\mathrm{Recover}}$ for recovering before the accident.

(5) $$\mathrm{Risk}=f(\frac{\mathrm{Probability of outcome}}{\mathrm{Acceptable probability of outcome}})=\log\left(\frac{q_{\mathrm{Trigger}}{\overset{¯}{B}}_{\mathrm{Avoid}}{\overset{¯}{B}}_{\mathrm{Recover}}}{P_{\mathrm{Acceptable}}(\mathrm{Outcome})}\right)$$

• Adopted by European Union Aviation Safety Agency (EASA) in European Risk Classification Scheme (ERCS) and Safety Risk Portfolio [6], [23]
• Electronic spreadsheet implementation of SIRA is available
• Explicitly models the barrier effectiveness
• Qualitative and quantitative versions

**Figure 7:**
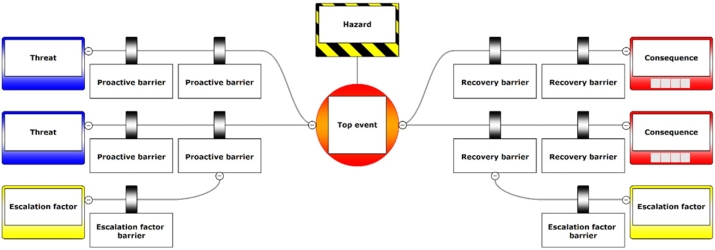
Example bowtie template with two threats, two most severe outcomes and multiple barriers.

*Bowtie.*  The bowtie model consists of different elements that build up and communicate a safety risk picture. A sample bowtie model structure is shown in Fig. 7.

• Used by UK CAA [5]
• Template models are available for exploration
• Qualitative assessment with detailed representation of threats, barriers and factors
• Could be used to support other methods

### 2.4 Risk management frameworks

A systematic approach to managing safety in aviation organizations is implemented through SMS [1], [2]. It involves all employees in the organization. A very compact and clear description of the SMS framework is provided by United Kingdom Overseas Territories Advisory Circular (UK OTAC) [3]

The standard safety management framework as defined by ICAO contains four components and twelve elements [1]:

• a safety policy and objectives (five elements)
• provision for safety risk management (SRM)
  **–** hazard identification
  **–** safety risk assessment and mitigation
• provision for safety assurance (SA)
  **–** safety performance monitoring
  **–** management of change
  **–** continuous improvement
• safety promotion
  **–** training and education
  **–** safety communication

Trainees are encouraged to discuss the SMS components and align them with the training material topics. Safety indicators for performance monitoring of specific activities can be proposed. SRM methodology can be selected based on organization size and operations complexity. Another practical exercise is to outline the contributions of each individual in a hypothetical organization needed for the proper operation of the system.

Safety reporting and investigation is an important part of the SMS, related to all components of the system. The ongoing process of reporting and investigation can be managed in the SA component of the framework. Applying an appropriate taxonomy can help the analyst to identify safety issues and monitor trends [2]. Safety policy should include safety reporting procedures as part of the management commitment element [1, Appendix 2, Sec. 1.1.1 c]. The safety reporting and investigation process is one of the main sources used to identify hazards. It is promoted as a separate element in the SRM component of the UK OTAC SMS framework [3].

## 3 Materials and methods

The aviation safety training methodology was used as part (module) of an aviation safety course for university students. The duration of this module of the course was two months. The workload was 5 hours per week including time for homework.

Students' knowledge was assessed at the end of the course through an eight-question questionnaire. Safety performance indicators participated with one question for calculation of an indicator and one for classification of events (both closed questions). Event sequence modelling was evaluated through two questions asking for event tree calculations and one question for checking model understanding. Safety concepts and SMS framework were tested by three open questions. The open questions required a short answer – a short paragraph or a list with just a few items. To reduce cheating, each question was randomly selected from three variants and the test duration was limited to 30 minutes.

Written exams from a previous version of the aviation safety course were reevaluated with the same scoring criteria. A control group of 47 student results was formed to establish baseline performance. The test group for the enhanced training methodology included 45 out of all 50 participants in the course. Five people were excluded from the survey because they missed the exam date and passed the exam later. Second attempts for score improvement were also ignored.

Each question was scored from zero to one point. A single score was calculated per student by summing the points for all questions. The maximum possible total score was eight. The minimum score was zero, but the expected value for random answers to closed-ended questions was 1.34. Regular statistics of the score (mean, variance, standard deviation, median and quantiles) were calculated for the test and the control groups.

The Mann-Whitney U test was used to compare the differences between the student groups as the scores were not normally distributed [24]. The distribution shapes were evaluated with Kolmogorov-Smirnov test for goodness of fit. The results for individual questions with a few possible answers were compared and assessed for significance by converting to contingency tables and using Pearson's chi-squared test of independence [25].

All exams were performed online through Chamilo learning management system (version 1.1x.xx, Chamilo Association, Lugo, Spain). All calculations were performed in Python (v.3.7.3) and Jupyter Notebook (v.6.1.4) environment with libraries Pandas 1.1.3, Scipy 1.5.2, Matplotlib 3.3.2 and Numpy 1.19.2. The study was approved by the Ethics Committee at the Academic Council of the Technical University of Sofia (Case 3/2021).

## 4 Results

The training methodology consists of three main tasks:

– introduction of basic concepts, notations and calculations;
– practice with simple examples;
– practice on industry developed data/models.

The methodology was applied following the theory and the models summarized in the previous sections. The ingredients of a safety management framework considered were:

– performance indicators
– accident scenarios
– risk management (hazards, risk events, outcomes, risk, metrics)

The effectiveness of the presented formal training in aviation safety was evaluated by comparing the results of two student classes denoted as Group 1 (“G1”) and Group 2 (“G2”). The topics, the problem sets and questionnaires were the same. The difference was the rigour in following the above training methodology with special emphasize on the notations and practice. For example, all performance indicators were expressed both in narrative and in symbolic forms. The students had first to define the conditional probabilities in P(Y|X) form and than to calculate both unconditional and conditional probabilities for different scenarios and end states. Reflection on the main components of risk was done for different risk assessment techniques and cases studies.

The presented methodology was followed strictly with G2 students. The main statistics in Table 5 demonstrate better performance with this group.

**Table 5:** Student score statistics by class.

	G1	G2
mean	4.67	5.36
std	2.03	1.16
min	0.50	2.90
25%	2.85	4.40
50%	4.60	5.50
75%	6.60	6.30
max	7.50	7.30

**Figure 8:**
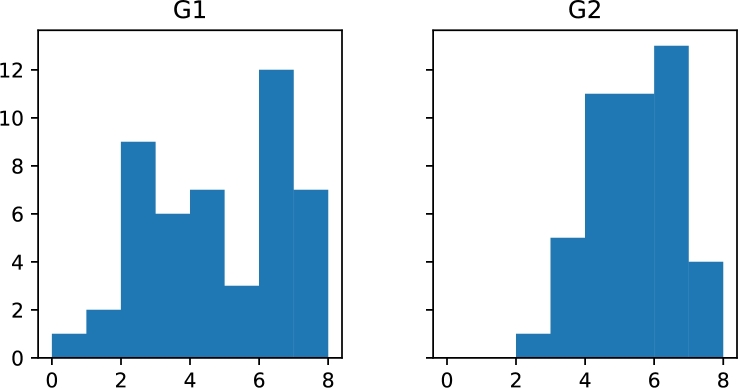
Histogram of student score by class. Group “G2” was trained in the proposed methodology.

A histogram of the total score is shown in Fig. 8. The most noticeable improvement was observed for low performing students. The significance of the change of the distribution was confirmed by Kolmogorov-Smirnov test (p-value=0.027).

The methodology was evaluated at topic level to identify areas with improved performance and areas that need further development. The results and their significance are:

• Event types and safety performance indicators – possible improvement, Mann-Whitney U test, p<0.10;
• Sequence of events, probabilities and outcomes – statistically significant improvement, Chi-square test, p<0.05;
• Safety concepts (hazard and risk) – no improvement was observed;
• Risk management frameworks – possible improvement, Mann-Whitney U test, p<0.10.

It is obvious that getting to grips with the qualitative part, i.e. discriminating the main concepts, is harder than the quantitative modelling of preset scenarios. The situation is complicated by the lack of consistent treatment and terminology in the literature. Therefore the safety concepts part of the methodology and it relation to practical implementations of the framework need to be enriched with more practice.

## 5 Discussion

The results of testing the safety training methodology are promising as the student engagement and average scores are improving. However, highlighting the nuances of the concepts can be expected only after gaining significant practical experience in applying the concepts to real cases.

Additional methods and techniques should be considered to support the future development of the methodology, e.g. system thinking based approaches such as the System Theoretic Accident Modelling and Process (STAMP) [26], the Functional Resonance Analysis Method (FRAM) [27] and resilience engineering. Integration of holistic techniques can be the subject of future development.

Aviation accidents are rare events. Most aviation organisations have no accidents. Therefore it is very easy to develop a wrong sense about the level of safety achieved and about safety management capabilities of the personnel. It is surprisingly hard to argue about safety related cases. For example, Regulation (EC) No 261/2004 relieve air carriers from obligations for compensation to passengers after delays in case of extraordinary circumstances, including “unexpected flight safety shortcomings” [28]. The application of the rule is left to lawyers and judges without further instructions and often without expert opinion.

Selecting the proper level of abstraction is challenging even for experts. Brooker points out improper selection of the relevant outcomes and consequences in the EUROCONTROL Safety Regulatory Requirements (ESARR 4) [10], [29]. Interpreting the numbers is also not trivial. Having safety targets with several significant digits is puzzling, given that quantifying even the order of the risk probability is a challenge (e.g. maximum tolerable probability for accident of 1.55e-8 per flight hour in [29]). Often the safety data and guidance materials are locked and inaccessible. Until the publication of Regulation (EU) 2020/2034 at the end of 2020 [30], it was difficult to find guidance on the ERCS metrics used since 2017 in the official annual safety reviews of EASA [6], [23].

A few questions related to the scope of the methodology can be discussed. Is the material too much? In the author's opinion it is not. Safety is an area where many people get a wrong impression of their competence. If the only result of the training is removing such complacency it could still be considered a success. Moreover, knowing the safety management workflow is important as safety is the responsibility of everyone.

Is the information too little? It depends. The methodology introduces the general principles of safety management. A separate training course should be provided to cover the specific expertise of the trainees, e.g. flight operations, airworthiness, airports, etc.

A number of safety related topics were not included in the training module, e.g. accident investigation, search and rescue, aviation regulations, human factors and aeronautical information. In addition, advanced hybrid causal models applicable for both tightly and loosely coupled system are available [31], [32], [33]. Such models stayed out of the scope of the current methodology as it should be accessible to a wide audience, but a training program developed for safety engineers can also include these.

## 6 Conclusion

A methodology for teaching the main concepts and models in aviation safety was introduced. Special emphasis was laid on the practical aspects of the concepts as notations, calculations and case studies. The presented approach has already demonstrated higher engagement and improved material mastery among students in aeronautical engineering.

Further studies are needed to improve identification of hazards, risks and performance metrics for specific domains and to develop corresponding student evaluation questionnaires. Understanding the abstract safety concepts continues to be the main challenge confronting students and practitioners.

## Declarations

### Author contribution statement

**K. Georgiev:** Conceived and designed the experiments; Performed the experiments; Analyzed and interpreted the data; Wrote the paper.

### Funding statement

This research did not receive any specific grant from funding agencies in the public, commercial, or not-for-profit sectors.

### Data availability statement

Data will be made available on request.

### Declaration of interests statement

The authors declare no conflict of interest.

### Additional information

No additional information is available for this paper.
